# Effect of Smartphone-Based Lifestyle Coaching App on Community-Dwelling Population With Moderate Metabolic Abnormalities: Randomized Controlled Trial

**DOI:** 10.2196/17435

**Published:** 2020-10-09

**Authors:** So Mi Jemma Cho, Jung Hyun Lee, Jee-Seon Shim, Hyungseon Yeom, Su Jin Lee, Yong Woo Jeon, Hyeon Chang Kim

**Affiliations:** 1 Department of Preventive Medicine Yonsei University College of Medicine Seoul Republic of Korea; 2 Department of Medicine the Graduate School of Yonsei University Seoul Republic of Korea; 3 Cardiovascular and Metabolic Diseases Etiology Research Center Yonsei University College of Medicine Seoul Republic of Korea; 4 Department of Internal Medicine Yonsei University College of Medicine Seoul Republic of Korea

**Keywords:** metabolic health, health behavior, lifestyle modification, mobile health

## Abstract

**Background:**

Metabolic disorders are established precursors to cardiovascular diseases, yet they can be readily prevented with sustained lifestyle modifications.

**Objective:**

We assessed the effectiveness of a smartphone-based weight management app on metabolic parameters in adults at high-risk, yet without physician diagnosis nor pharmacological treatment for metabolic syndrome, in a community setting.

**Methods:**

In this 3-arm parallel-group, single-blind, randomized controlled trial, we recruited participants aged 30 to 59 years with at least 2 conditions defined by the Third Report of the National Cholesterol Education Program expert panel (abdominal obesity, high blood pressure, high triglycerides, low high-density lipoprotein cholesterol, and high fasting glucose level). Participants were randomly assigned (1:1:1) by block randomization to either the nonuser group (control), the app-based diet and exercise self-logging group (app only), or the app-based self-logging and personalized coaching from professional dieticians and exercise coordinators group (app with personalized coaching). Assessments were performed at baseline, week 6, week 12, and week 24. The primary outcome was change in systolic blood pressure (between baseline and follow-up assessments). Secondary outcomes were changes in diastolic blood pressure, body weight, body fat mass, waist circumference, homeostatic model of assessment of insulin resistance, triglyceride level, and high-density lipoprotein cholesterol level between baseline and follow-up assessments. Analysis was performed using intention-to-treat.

**Results:**

Between October 28, 2017 and May 28, 2018, 160 participants participated in the baseline screening examination. Participants (129/160, 80.6%) who satisfied the eligibility criteria were assigned to control (n=41), app only (n=45), or app with personalized coaching (n=43) group. In each group, systolic blood pressure showed decreasing trends from baseline (control: mean –10.95, SD 2.09 mmHg; app only: mean –7.29, SD 1.83 mmHg; app with personalized coaching: mean –7.19, SD 1.66 mmHg), yet without significant difference among the groups (app only: *P*=.19; app with personalized coaching: *P*=.16). Instead, those in the app with personalized coaching group had greater body weight reductions (control: mean –0.12, SD 0.30 kg; app only: mean –0.35, SD 0.36 kg, *P*=.67; app with personalized coaching: mean –0.96, SD 0.37 kg; *P*=.08), specifically by body fat mass reduction (control: mean –0.13, SD 0.34 kg; app only: mean –0.64, SD 0.38 kg, *P*=.22; app with personalized coaching: mean –0.79, SD 0.38 kg; *P*=.08).

**Conclusions:**

Simultaneous diet and exercise self-logging and persistent lifestyle modification coaching were ineffective in lowering systolic blood pressure but effective in losing weight and reducing body fat mass. These results warrant future implementation studies of similar models of care on a broader scale in the context of primary prevention.

**Trial Registration:**

ClinicalTrials.gov NCT03300271; http://clinicaltrials.gov/ct2/show/NCT03300271

## Introduction

Metabolic disorders are established precursors to cardiovascular disease [1]. Previous studies have demonstrated that even at subclinical stages, persons with elevated blood pressure, blood glucose level, cholesterol level, and adiposity are at significantly higher risk of adverse cardiometabolic outcomes [2]. Despite recent health care policy changes that have expanded healthy lifestyle advocacy initiatives, a considerable proportion do not achieve the guideline-recommended metabolic profile [3-5]. Therefore, timely and persistent management of metabolic abnormalities are crucial in preventing adverse health outcomes at an individual level and conserving substantial health care costs at a national level.

Alongside the advent of new medicines, technological innovations have aided in easing accessibility to and enriching the quality of health care. They have eliminated practical barriers, thereby allowing the distribution and improvement of health care via nonconventional routes at unprecedented speeds [6]. Several features include the transmission of medical records, social media forums for open discussion, web-based interactive education programs, higher precision diagnostics, real-time status tracking, digitalized clinics (ie, telemonitoring), and prescription dispensation [7,8].

In particular, the ubiquity of mobile phone technology has incentivized industries to create smartphone-based apps for health monitoring [9]. Previous trials [10,11] have evaluated the efficacy and effectiveness of such tools in the context of secondary prevention. For example, among patients receiving cardiac rehabilitation after hospitalization for myocardial infarction, daily text message reminders led to greater medication adherence and exercise capacity compared with patients receiving usual care [10]. The utility extends to general populations; a Finnish trial [11] demonstrated both short- and long-term weight loss among people who were overweight and who logged weight daily and received dietary management instructions over a period of 1 year. By collecting data in real time, these mobile-based apps enable researchers to assess multiple behaviors and to prompt change at low cost and with high ease.

Nonetheless, there are important limitations in the current literature. Previous studies [12-14] have primarily recruited clinic patients who were already using or were exceptionally motivated to use health management tools. Methodologically, many studies [7] estimated the effect of these mobile interventions based on per protocol analysis. It has been established that physical activity and diet affect blood pressure level not only in hypertension patients but also in people with prehypertension or elevated blood pressure [15]. Recent blood pressure guidelines [15,16] emphasized early lifestyle modification for people whose blood pressure is above the normal range. However, the effects of smartphone-based apps in lowering blood pressure have not been properly assessed in people whose blood pressure is above the normal range in real-world settings.

In this context, the objective of the study was to evaluate the longitudinal effect of smartphone-based health care app on metabolic parameters in a sample of the general population with moderate metabolic abnormalities yet without clinical diagnosis nor pharmacological treatment. We hypothesized that the participants receiving both real-time personalized coaching and self-logging diet and physical activity would yield greater improvements in blood pressure and other metabolic parameters than those who were only self-logging or who did not use the app.

## Methods

### Study Design

The study was designed as a single-blind 3-arm parallel-design randomized controlled trial delivering a 6-month primary prevention program via mobile app to a population with moderate metabolic abnormalities with neither diagnosis nor treatment for metabolic disorders. The main objective was to evaluate the effectiveness of health management app on metabolic parameters over 3 follow-up examinations. Community-dwelling adults residing in Seoul and nearby capital regions were recruited. The study protocol was approved by the institutional review boards of Severance Hospital and Yonsei University Health System (4-2017-0666), and the protocol of the study is registered at ClinicalTrials.gov (NCT03300271).

### Participants

Candidate participants were identified based on objectively measured metabolic profiles from a previous Cardiovascular and Metabolic Diseases Etiology Research Center (CMERC) observational cohort study [17]. Briefly, the CMERC study [17] aimed to identify novel risk factors and to investigate the distribution and effects of known cardiovascular and metabolic diseases risk factors.

We recruited smartphone users aged 30-59 years with at least 2 metabolic abnormalities defined by the modified version of the Third Report of the National Cholesterol Education Program expert panel on detection, evaluation, and treatment of high blood cholesterol in adults [18] using the criteria for Asian populations: waist circumference (male: ≥90 cm; female: ≥80 cm), blood pressure (systolic: ≥135 mmHg; diastolic: ≥85 mmHg), triglyceride ≥150 mg/dL, high-density lipoprotein (HDL) cholesterol (male: <40 mg/dL; female: <50 mg/dL), and fasting glucose level ≥100 mg/dL. Exclusion criteria included users of smartphone-based health care apps for lifestyle modification, individuals with a previous diagnosis of cardiovascular disease, malignant cancer, or metabolic syndrome, individuals who were taking antihypertensives, lipid- or glucose-lowering drugs, and women who were pregnant or breastfeeding at the time of the study.

**Figure 1:**
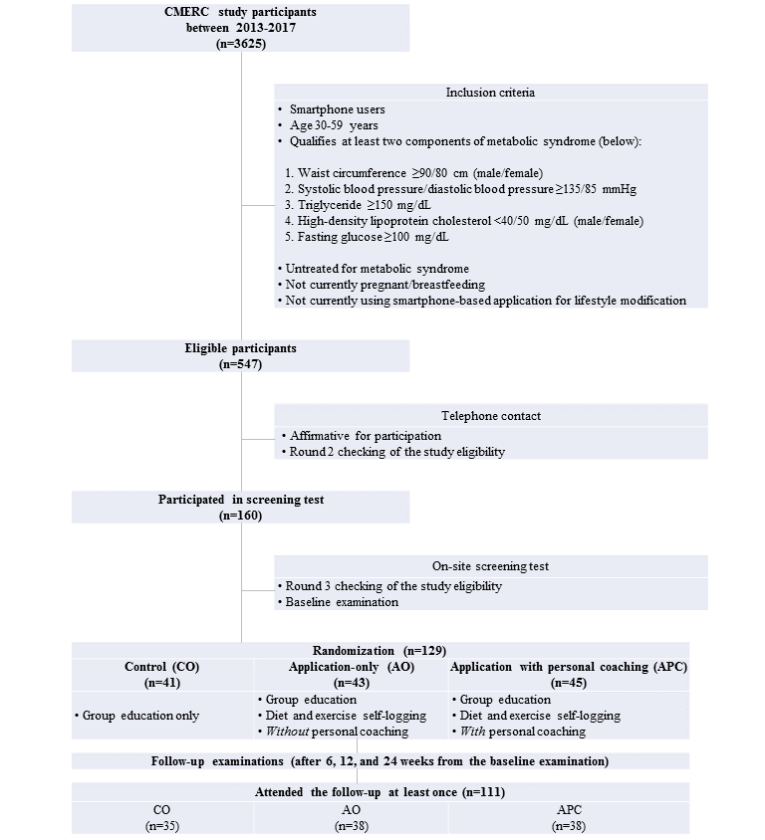
Flow diagram of the study participants. CMERC: Cardiovascular and Metabolic Diseases Etiology Research Center.

Among 3625 CMERC cohort participants [17], 546 people qualified. We contacted these individuals via telephone and mail for recruitment. Of the 160 (29.1%) who expressed affirmative for screening test, a total of 129 participants (23.6%) attended the baseline examination (Figure 1).

Participants were asked to give their written consent without knowledge of the intervention assignments.

### Randomization and Masking

We randomly allocated participants into 1 of the 3 intervention arms via sex- and age-stratified block randomization. Specifically, we set the block size equal to 6 from sex (male or female) by age group in decile (30, 40, and 50) using R (version 3.4.4; R Foundation for Statistical Computing). We adopted a single-blind approach; thus, the effectiveness would be assessed by masked researchers unaware of the randomization results. The allocation concealment was achieved via individualized texting, which instructed all participants to adhere to their respective intervention, to avoid using other health management apps, and to refrain from sharing of their intervention instructions with each other for the entire study duration. To minimize crossover or contamination, we (1) retrospectively ensured no unallocated feature had been installed to the app only group from the app’s user-specific metadata and (2) asked the participants whether they had engaged in other health-related trials, programs, or apps at every follow-up assessment.

### Procedures

**Figure 2:**
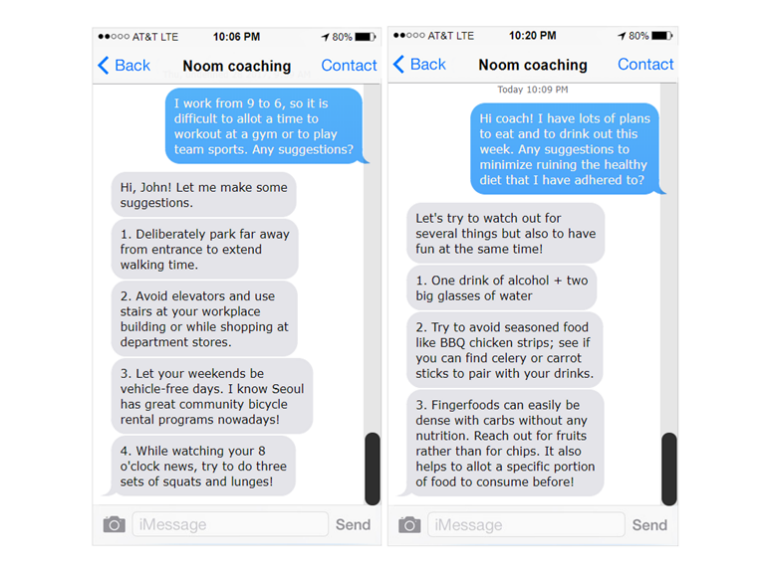
Example of personalized coaching.

At the baseline assessment, all participants received on-site education that entailed information on metabolic syndrome and preventive strategies, including validated exercise regimens and nutritious cooking recipes. Then, we randomly allocated participants into 1 of the 3 intervention arms: the control group (control; n=41) received only the aforementioned baseline education and was asked to refrain from concurrently engaging in any smartphone-based lifestyle modification app or programs; the app only (app only; n=45) and the app with personalized coaching (app with personalized coaching; n=43) groups also received the baseline education and were additionally asked to use a smartphone-based weight management app called Noom (Noom Inc). Specifically, Noom allows users to log details regarding daily food intake and physical activities. For instance, a user can record a specific menu item (ie, fastfood chain M’s cheeseburger), consumed portion (ie, single serving, half of cheeseburger), and time of consumption (ie, breakfast, 8 AM). From Noom’s nutrition metadata, the app readily calculates total calories and macronutrients consumed for each log entry. Likewise, a wide array of physical activities (ie, indoor treadmill walking) are available for selection from the predefined list. After entering the intensity (ie, pace) and duration (ie, 60 minutes) of physical activity, the app yields energy expenditure in kilocalories, accounting for the user’s sex, age, height, and weight. As an additional feature, it delivers structured health-related curricula and personalized feedback from certified exercise regimen coordinators and clinical dieticians based on their reviews of the user’s logs (Figure 2).

The default frequency of personalized coaching was set to 3 times per week; however, the actual frequency varied depending on the user’s participation rate. Only the app with personalized coaching group received the personalized coaching. All users’ activation statuses were evaluated based on the number of weight, meal, and physical activity logs. Weekly, we defined each user as active if the user had recorded at least 1 aforementioned parameter.

The universal baseline examination included anthropometric measurements, blood tests, and face-to-face interviews on demographics, disease history, and health behavior. Then, participants received text messages notifying them of the study initiation. The app only and app with personalized coaching groups were given additional instructions regarding the Noom app installation procedures; each user received a unique identification code.

### Outcomes

The primary outcome was change in systolic blood pressure between the baseline and follow-up at 6, 12, and 24 weeks. The secondary outcomes were changes in diastolic blood pressure, body weight, body fat mass, waist circumference, homeostatic model of assessment of insulin resistance (HOMA-IR), and lipid profile (triglyceride and HDL cholesterol) between the baseline and follow-up at 6, 12, and 24 weeks.

Anthropometric measurements were performed with strict adherence to standardized protocols and using calibrated equipment. Blood pressure was consecutively measured using both single- and double-arm automated oscillometric devices (HEM-7080, Omron Health; WatchBP Office Central, Microlife) at a single sitting. The mean of second and third measurements were used for analysis. Participants underwent blood tests after overnight fasting for a minimum of 8 hours. Fasting plasma glucose and insulin levels were assessed using colorimetry method (ADVIA1800 Auto Analyzer, Siemens Medical Solutions). HOMA-IR was calculated as the product of fasting glucose and insulin levels divided by 405 in mg/dL. Weight was measured to the nearest 0.1 kg on a digital scale (DB-150, CAS). To minimize measurement variability, a zero-point adjustment was routinely conducted using weight blocks (20, 40, and 60 kg). Bioelectrical impedance analysis delineated body composition (BSM-330, INBODY). Waist circumference was measured to the nearest 0.1 cm using a plastic tape (SECA 201, SECA), while maintaining the level of the measuring tape.

Physical activity was assessed by the Korean version of the International Physical Activity Questionnaire [19]. Using validated alcohol consumption and cigarette smoking ratings, participants were divided into non-, previous-, and current categories for alcohol consumption and smoking.

### Statistical Analysis

The target sample size was 150 participants, chosen to provide precise estimates of the intended effect of mobile app usage. Specifically, the sample size calculation was conducted based on the expected difference in systolic blood pressure after 24 weeks across the 3 intervention arms [20]. We assumed a statistical power of 80% and a significance level of *P*<.05. Based on previous literature [20,21], we expected a mean systolic blood pressure difference of 6 mmHg with a standard deviation of 10 mmHg after 24 weeks. When considering 10% attrition rate and 3-arm design, the study required a minimum recruitment of 150 participants.

We employed analysis of variance to assess differences in demographic and health-related behavior. Then, we compared the extent of changes in each metabolic parameter across participants randomly assigned to control versus app only and app with personalized coaching groups, separately, using an intention-to-treat approach; all participants who participated in at least 1 of the 3 follow-up assessment were included in the analysis. We used independent *t* tests to evaluate the primary and secondary outcomes at each time point. To account for repeated measurements over multiple follow-ups, we employed a linear mixed model to determine the effect of mobile health care apps on the prespecified outcomes. Specifically, the unstructured linear mixed model incorporates time and group×time interaction terms to assume no homogeneity across the 3 groups at the baseline. From the random intercept model, the estimated beta coefficient of the group×time interaction term was regarded as the effect of the app usage. The changes are presented as estimated beta coefficient (β) and standard error (SE). All statistical tests were 2-sided and the statistical significance was set at a *P*<.05. All analyses were performed using R and SAS (version 9.4; SAS Institute Inc).

All data were collected, registered, and managed on ClinicalTrials.gov (NCT03300271). Data were deidentified and accessible only by designated researchers. All researchers strictly adhered to data security protocols. For unbiased data monitoring and trial safety overseeing, the research director delegated Dae Ryong Kang, a professor of biomedical data science at Yonsei University, Wonju College of Medicine, Wonju, Korea, who remained independent of the research execution.

## Results

### Participants

All participants were enrolled on October 28, 2017, and the last participant completed the week 24 follow-up on June 2, 2018. Of the 129 enrolled individuals, 41 (32%) were randomly assigned to the control group, 45 (35%) to the app only group, and 43 (33%) to the app with personalized coaching group (Figure 1). In the end, 111 participants attended at least 1 follow-up examination (week 6: 107; week 12: 100; week 24: 105), yielding a 14.0% attrition rate overall.

### Baseline Characteristics

Table 1 shows the general characteristics of the study participants at the baseline screening. Overall, the participants were similarly distributed in terms of age, metabolic parameters, and health behaviors across the 3 groups. At baseline, systolic blood pressure was comparable across the 3 groups (control: mean 131.8 mmHg; app only: mean 130.8 mmHg; app with personalized coaching: mean 133.3 mmHg). Such comparability across the groups ensured the baseline differences did not affect the changes in metabolic parameter over the study period.

**Table 1:** General characteristics of the study participants at the baseline screening.

Variables		Control (n=41)	App only (n=45)	App+personalized coaching (n=43)
Age (years), mean (SD)		49.5 (7.9)	49.2 (7.5)	48.9 (7.8)
**Sex, n (%)**				
	Male	19 (46.3)	23 (51.1)	21 (48.8)
	Female	22 (53.7)	22 (49.9)	22 (51.2)
Number of metabolic abnormalities, mean (SD)		2.9 (0.8)	2.9 (0.9)	2.8 (0.9)
Systolic blood pressure (mmHg), mean (SD)		131.8 (15.8)	130.8 (15.2)	133.3 (14.9)
Diastolic blood pressure (mmHg) , mean (SD)		87.4 (9.9)	86.6 (10.7)	89.0 (11.7)
Height (cm), mean (SD)		166.4 (8.8)	165.3 (10.4)	164.0 (8.9)
Weight (kg, mean (SD)		71.8 (13.2)	72.6 (12.4)	71.9 (11.6)
Body mass index (kg/m^2^), mean (SD)		25.8 (2.8)	26.5 (3.2)	26.6 (2.9)
Waist circumference (cm) , mean (SD)		89.1 (8.2)	90.8 (8.2)	90.4 (7.0)
Percent body fat, mean (SD)		30.3 (6.8)	31.9 (7.1)	31.6 (5.7)
Fat mass (kg), mean (SD)		21.5 (5.2)	23.1 (6.4)	22.5 (4.5)
Skeletal muscle mass (kg) , mean (SD)		28.1 (7.1)	27.6 (6.2)	27.5 (6.1)
Total cholesterol (mg/dL), mean (SD)		212.9 (31.6)	197.4 (34.1)	205.1 (31.8)
Triglyceride (mg/dL), mean (SD)		212.9 (138.0)	176.6 (107.7)	169.2 (72.7)
HDL^a^ cholesterol (mg/dL), mean (SD)		46.1 (8.4)	47.1 (11.2)	46.3 (10.5)
LDL^b^ cholesterol (mg/dL), mean (SD)		139.1 (28.8)	127.5 (35.0)	137.1 (30.7)
Fasting blood glucose (mg/dL), mean (SD)		98.1 (13.8)	105.9 (39.4)	98.9 (19.0)
Insulin (mIU/mL), mean (SD)		11.4 (4.8)	11.5 (4.8)	11.8 (3.9)
Hemoglobin A_1c_ (%), mean (SD)		5.9 (0.7)	6.1 (1.2)	5.8 (0.7)
HOMA-IR^c^, mean (SD)		2.8 (1.2)	3.0 (1.5)	2.9 (1.5)
Hypertension, mean (SD)		2 (4.9)	2 (4.4)	1 (2.3)
Diabetes, mean (SD)		0 (0.0)	0 (0.0)	0 (0.0)
Dyslipidemia, mean (SD)		3 (7.3)	1 (2.2)	1 (2.3)
**Smoking status, n (%)**				
	Never	19 (46.3)	28 (62.2)	25 (58.1)
	Former	8 (19.5)	11 (24.4)	11 (25.6)
	Current	14 (34.2)	6 (13.3)	7 (16.3)
**Alcohol consumption, n (%)**				
	Never	5 (12.2)	8 (17.8)	5 (11.6)
	Former	2 (4.9)	0 (0.0)	2 (4.7)
	Current	34 (82.9)	37 (82.2)	36 (83.7)
Alcohol consumption (g/day), mean (SD)		22.1 (38.8)	10.4 (15.5)	11.5 (18.1)
**MVPA^d^ engagement, n (%)**				
	Yes	2 (4.9)	8 (17.8)	9 (20.9)
	No	39 (95.1)	37 (82.2)	34 (79.1)
Sedentary time (hours/day), mean (SD)		7.2 (2.7)	6.6 (2.8)	7.1 (3.9)

^a^HDL: high-density lipoprotein.

^b^LDL: low-density lipoprotein.

^c^HOMA-IR: homeostatic model assessment of insulin resistance.

^d^MVPA: moderate-vigorous physical activity.

To ensure homogeneity between those who attended and those who did not attend follow-up at least once, we have compared the baseline characteristics as illustrated in Multimedia Appendix 1. In regard to age, sex, anthropometry, and glycemic and lipid profiles, no statistically significant differences (age: *P*=.10; sex: *P*=.09; systolic blood pressure: *P*=.36; diastolic blood pressure: *P*=.93; BMI: *P*=.98; total cholesterol: *P*=.95; fasting glucose level: *P*=.25) were detected between the 2 groups. The only notable difference was the proportion of current smokers; those who did not attend any follow-up examinations had a higher proportion of current smokers than those who attended at least once (44.4% versus 17.1%; *P*=.04; Multimedia Appendix 2).

### Comparisons at Each Time Point

**Figure 3:**
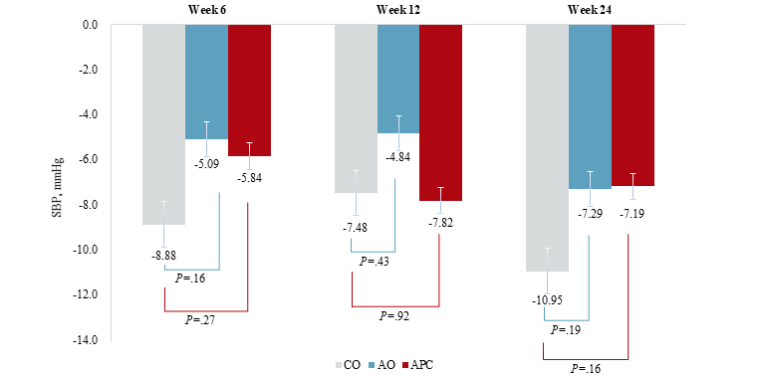
Systolic blood pressure changes from the baseline across the 3 groups at each time point. AO: app-only; APC: app with personalized coaching; CO: control, SBP: systolic blood pressure.

All 3 groups showed varying amounts of systolic blood pressure reduction overall. In reference to the baseline examination, the control and app only groups showed the most dramatic reduction at week 24 (control: mean –10.95, SD 11.98 mmHg; app only: mean –7.19, SD 9.98 mmHg), whereas the app with personalized coaching group showed the most dramatic systolic blood pressure reduction at week 12 (mean –7.82, SD 11.98 mmHg). However, compared to the control group, neither the app only nor the app with personalized coaching group had significantly different systolic blood pressure change at any follow-up examination (Figure 3).

**Figure 4:**
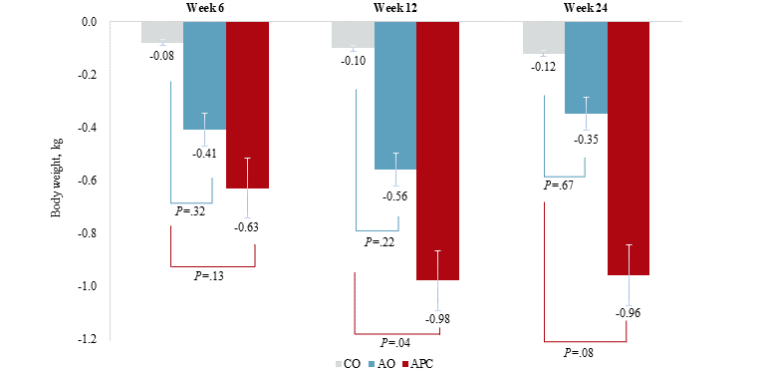
Body weight changes from the baseline across the 3 groups at each time point. AO: app-only; APC: app with personalized coaching; CO: control, SBP: systolic blood pressure.

**Figure 5:**
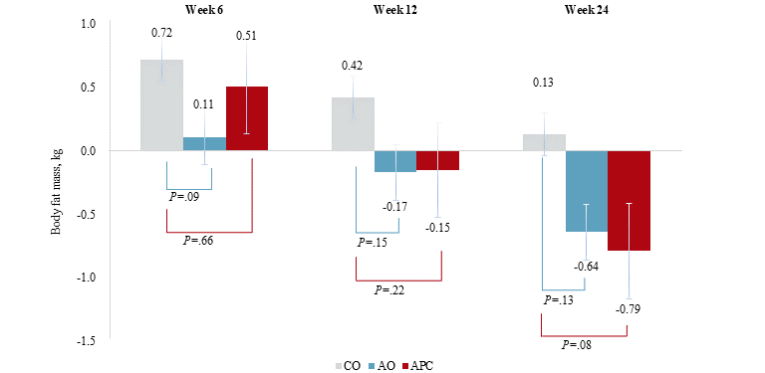
Body fat mass changes from the baseline across the 3 groups at each time point. AO: app-only; APC: app with personalized coaching; CO: control.

For secondary outcomes, participants in the app with personalized coaching group generally lost more body weight (mean –0.96 kg versus –0.12 kg; Figure 4), lost more body fat mass (mean –0.79 kg versus 0.13 kg; Figure 5), and had smaller waist circumferences (mean –1.86 cm versus –0.08 cm; Multimedia Appendix 2) by the end of the study period. At week 24, diastolic blood pressure (*P*=.42; Multimedia Appendix 3) and other glycemic (fasting glucose level: *P*=.99; HOMA-IR: *P*=.63; Multimedia Appendix 4 and Multimedia Appendix 5, respectively), and lipid indices (triglyceride level: *P*=.93; HDL cholesterol level: *P*=.46; Multimedia Appendix 6 and Multimedia Appendix 7, respectively) had not changed significantly.

### Linear Mixed Model

Neither the app only (control versus app only at week 24: β=2.93, SE 2.42, *P*=.23) nor the app and additional personalized feedback feature (control versus app with personalized coaching at week 24: β=3.18, SE 2.42, *P*=.19) significantly contributed to systolic blood pressure reduction at any of the follow-up examinations (Multimedia Appendix 1). In regard to secondary outcomes, statistically significant effects were observed for body weight and body fat. Compared to the participants in the control group, those in the app with personalized coaching group lost more body weight at week 12 (β=–0.93, SE 0.43; *P*=.03) and at week 24 (β=–0.87, SE 0.42; *P*=.04). This translated to an average difference of 0.14 kg of body weight loss per month between participants in the control group and participants in the app with personalized coaching group. In parallel, the app with personalized coaching group lost more body fat than the control group did at week 24 (β=–0.95, SE 0.46, *P*=.04). This was equivalent to participants in the app with personalized coaching group losing an average of 0.19 kg more body fat per month than those in the control group. The app with personalized coaching group showed significant HOMA-IR level reductions at week 12 (β=–0.55, SE 0.27, *P*=.04). However, separate examination by fasting glucose level did not show significance (*P*=.80) and the HOMA-IR reductions diminished by week 24 (β=–0.22, SE 0.26, *P*=.40). Otherwise, there were no significant effects of the app on diastolic blood pressure (app only: *P*=.83; app with personalized coaching group: *P*=.47), waist circumference (app only: *P*=.32; app with personalized coaching: *P*=.12), triglyceride (app only: *P*=.70; app with personalized coaching: *P*=.23), HDL cholesterol (app only: *P*=.14; app with personalized coaching: *P*=.91), or fasting blood glucose level (app only: *P*=.38; app with personalized coaching: *P*=.84; Multimedia Appendix 1).

## Discussion

### Principal Findings

Mobile-based behavioral health interventions that permit real-time data collection and sharing are increasingly commonplace, enabling researchers to assess multiple health behaviors in various contexts. At the same time, they prompt users to be more self-aware and to modify their own health behavior. In this context, the goal behind this study was to assess the real-world effectiveness of a smartphone-based self-monitoring health management app in a community-dwelling population of individuals without diagnosis or treatment of metabolic disorders. App usage showed differential effects on metabolic parameters at different time points. Overall, the primary outcome of systolic blood pressure followed a decreasing trend from the baseline yet did not change notably between the 3 groups at any follow-up examinations. Instead, the simultaneous diet/exercise logging and lifestyle coaching yielded relatively greater body weight reduction, specifically via body fat mass reduction. These effects were attenuated yet sustained at 6 month.

Despite no significant systolic blood pressure improvement in this study, previous studies [12-14,22-24] have identified the utility of mobile apps in lowering blood pressure through self-monitoring and information delivery services. In several randomized controlled trials [12-14,22-24] conducted in obesity clinic settings, text messages or emails for antihypertensive medication adherence, tailored guidance on salt intake, smoking cessation, and physical activity interventions reported significant differences in blood pressure reduction compared to usual care. However, unlike our study in which community-dwelling individuals without overt metabolic syndrome participated, similar studies [7,21,25] have been primarily performed on patients based on completers’ analysis rather than intention-to-treat. Considering varied levels of initiative for health management often used as a proxy for adherence and attrition, our results indicate that effectiveness of these self-care apps may considerably differ by study population and analytical methods.

Moreover, Liu and colleagues’ [25] examination of internet-based counseling interventions among patients with elevated blood pressure indicated that e-counseling interventions significantly reduced daytime systolic blood pressure by 3.8 mmHg (95% CI −5.63 to −2.06), with greater reductions from more sustained interventions. Since our study relied on a single-occasion blood pressure measurement in the examination setting, the results may potentially be distorted by whitecoat or masked hypertension.

Furthermore, a significant limitation of accumulated evidence is that many studies [7] were conducted for durations less than 6 months. Given that hypertension is a chronic condition that requires long-term pharmacological treatment and lifestyle modification, prolonged observation should be allotted for more accurate assessment of intervention adherence and subsequent blood pressure changes.

In our study, body weight reduction, via body fat mass loss, was the most evident improvement from app usage, as indicated by similar trials [26,27]. Among overweight adults, daily transmitted personalized multimedia message services providing weight control materials have proven to induce greater weight loss (−1.97 kg difference, 95% CI −0.34 to −3.60 kg) than the non-receiving group [26]. A systematic review [25] consistently identified the association between self-monitoring or online obesity treatment programs and body size reduction via targeted advice on reduced energy intake, increased physical activity, and social support. Often, the studies incorporated the use of structured regimen, regular self-monitoring, circumstantially appropriate feedback, prompt communication, and social support [27]. Abraham et al [28] classified such intervention content, including information-motivation-behavior skills model (ie, providing information about behavior-health link), social-cognitive theory (ie, prompting barrier identification, general encouragement), control therapy (ie, self-monitoring, interactive feedback), relapse prevention and behavior sustenance, and more. Although the extent and nature of the interventions varied across studies, these elements commonly found in mobile technology interventions, altogether, appear crucial in driving greater changes.

Our study results indicated that the 6-month usage of the mobile app did not substantially improve insulin sensitivity. Yet, an analogous meta-analysis [29] demonstrated that comprehensive lifestyle modification delivered through a mobile app lowered hemoglobin A_1c_ level by an average of 0.5% over 6 months of follow-up. Interestingly, this decrease was not accompanied by concurrent improvements in other diabetes risk factors, such as blood pressure, cholesterol levels, or adiposity [29]. However, considering that hemoglobin A_1c_ reflects long-term fluctuations of glycemic control (unlike fasting glucose level used in our study), Liang et al [29] raised concerns regarding insufficient follow-up period. Moreover, since these trials were conducted on persons diagnosed with diabetes of varying subtypes and adherence to antihyperglycemic treatments, it would have been challenging to isolate the effectiveness of the mobile app intervention from changes in treatment behavior. Therefore, the different representation of glycemic state and participant characteristics warrant caution in assuming or denying the attribution of these technology-based intervention to other nonspecific benefits.

Over the span of 6 months, our participants did not show significantly different changes in triglyceride and HDL cholesterol levels across the 3 groups. Yet, Park and Kim [30] showed that, among gynecology and family medicine outpatients, the use of web-based diet and exercise diaries and the specialists’ weekly lifestyle modification recommendations improved total (–12.9 mg/dL) and low-density lipoprotein (–11.3 mg/dL) cholesterol levels after 12 weeks. Again, it is hard to extrapolate from clinic setting population in which individuals may already be utilizing health management app prior to trial entrance or may be taking medications (ie, statins) that distort the true effects of the interventions.

The greatest novelty of our study lies in its evaluation of the mobile app’s effectiveness via intention-to-treat approach in nonpatient population without the concern of confounding by pharmacological treatment. Considering that the general population consists of individuals with wide-ranging levels of digital literacy and utility, willingness for lifestyle modification, and physiological and socioeconomic backgrounds, our study portrays the effectiveness of mobile-based health management app in real-world setting. Furthermore, the assessor-blinding enabled true compliance assessment in the sample regardless of individual preference for or competency with the app, thereby evaluating the app’s real-world potential. In addition, because we assessed the changes in metabolic parameters repeatedly over a considerable time horizon, our results reflect the long-term trajectory of the metabolic indices, empirically. Lastly, as a 3-arm parallel design with concurrent control, we were able to discriminate whether the self-logging of diet and exercise was sufficient to induce improvements in metabolic parameter or whether additional personalized coaching was the critical element in shaping healthy behavior.

Nonetheless, several limitations warrant cautious interpretation of our study findings. During the screening test, we faced an unexpectedly greater number of participants who did not satisfy the inclusion criteria, and thus, we were unable to reach the goal sample size. During the study period, because the participants had varying attendance to each of the follow-up examination, there may have been residual heterogeneity among participants who attended follow-up examinations none, once, twice, or all follow-up examinations. Yet, those lost to follow-up were demographically similar to those who participated in the study with the exception of current smoking status. If any effects were present, we expect such attrition to have an effect on the study results toward the null. Moreover, because the participants were selected from an already-established cohort, higher self-efficacy and proficiency may have been present. Therefore, the results may not be entirely generalizable to the general population. However, the original cohort study was purely observational; thus, our findings still represent individuals typically seeking health management without referral to physician nor pharmacological treatment. Furthermore, similar literature has suggested that baseline self-efficacy appraisals may not be entirely pertinent to practical skills or opportunities to sustain life changes [15]. In this context, we expect comparable initiatives for lifestyle modification between our study and the general population. Lastly, considering that blood pressure, body size, glycemic index, and lipid indices are reflective of chronic states, our 6-month study period may have been insufficient to witness changes. Future studies with a larger sample size and a prolonged study period may better assess the effectiveness of lifestyle modification mobile apps on the long-term trajectory of metabolic indices.

### Conclusions

Among the community-dwelling adults with moderate metabolic abnormalities without diagnosis or treatment for disorders, we examined the effect of a smartphone-based app on changes in metabolic parameters. By the end of the 6-month study period, no changes to systolic blood pressure were significant for participants who utilized both diet/exercise logging and personalized coaching compared to logging-only or nonusing groups. Instead, the self-logging and lifestyle coaching yielded greater body weight reduction via body fat mass loss. The research investigating mobile health management interventions that confer accessible and cost-effective benefits remains in its infancy, especially in general populations. Future studies focusing on comparative effectiveness using alternative study designs and on populations of various health, socioeconomic, and cultural backgrounds are needed to integrate these apps in everyday lives and clinic practice.

## Appendix

Multimedia Appendix 1 Supplementary tables.

Multimedia Appendix 2 Diastolic blood pressure changes from the baseline across the 3 groups at each time point. AO: app-only; APC: app with personalized coaching; CO: control.

Multimedia Appendix 3 Waist circumference changes from the baseline across the 3 groups at each time point. AO: app-only; APC: app with personalized coaching; CO: control.

Multimedia Appendix 4 Fasting glucose level changes from the baseline across the 3 groups at each time point. AO: app-only; APC: app with personalized coaching; CO: control.

Multimedia Appendix 5 Homeostatic model of assessment of insulin resistance changes from the baseline across the 3 groups at each time point. AO: app-only; APC: app with personalized coaching; CO: control.

Multimedia Appendix 6 Triglyceride level changes from the baseline across the 3 groups at each time point. AO: app-only; APC: app with personalized coaching; CO: control.

Multimedia Appendix 7 High-density lipoprotein cholesterol level changes from the baseline across the 3 groups at each time point. AO: app-only; APC: app with personalized coaching; CO: control.

Multimedia Appendix 8 CONSORT-eHEALTH checklist (V 1.6.1).

## Abbreviations

CMERC: Cardiovascular and Metabolic Diseases Etiology Research Center
HDL: high-density lipoprotein
HOMA-IR: homeostatic model of assessment of insulin resistance
