# Supplementary material for: Effect of Smartphone-Based Lifestyle Coaching App on Community-Dwelling Population With Moderate Metabolic Abnormalities: Randomized Controlled Trial
Source: J Med Internet Res. 2020 Oct 9;22(10):e17435. doi: 10.2196/17435 (PMC7584978; doi:10.2196/17435)
Supplement: Multimedia Appendix 1 [file jmir_v22i10e17435_app1.docx]

| Supplementary Table 1. Comparison of general characteristics between participants who have | | | | | | | | | |
| --- | --- | --- | --- | --- | --- | --- | --- | --- | --- |
| and have not attended single follow-up examination. | | | | | | | | | |
| Variables | Attended follow-up | | |  | Did not attend follow-up | | |  | *^d^P* value |
|  |  |  |  |  |  |  |  |  |  |
|  | (n=111) | | |  | (n=18) | | |  |  |
| Age, year | 49.6 | ± | 7.7 |  | 46.4 | ± | 7.5 |  | 0.10 |
| Sex |  |  |  |  |  |  |  |  | 0.09 |
| Male | 63 |  | (47.7) |  | 13 |  | (72.2) |  |  |
| Female | 48 |  | (52.3) |  | 5 |  | (27.8) |  |  |
| No. metabolic abnormalities | 3.2 | ± | 0.8 |  | 3.4 | ± | 0.8 |  | 0.89 |
| Systolic blood pressure, mmHg | 132.5 | ± | 15.4 |  | 128.9 | ± | 14.3 |  | 0.36 |
| Diastolic blood pressure, mmHg | 87.6 | ± | 11.1 |  | 87.9 | ± | 8.4 |  | 0.93 |
| Height, cm | 164.7 | ± | 9.2 |  | 168.5 | ± | 10.2 |  | 0.11 |
| Weight, kg | 71.6 | ± | 12.2 |  | 75.1 | ± | 12.8 |  | 0.26 |
| Body mass index, kg/m^2^ | 26.3 | ± | 3.0 |  | 26.3 | ± | 3.0 |  | 0.98 |
| Waist circumference, cm | 89.8 | ± | 7.9 |  | 91.8 | ± | 7.0 |  | 0.31 |
| Fat mass, kg | 22.5 | ± | 5.6 |  | 21.8 | ± | 4.5 |  | 0.63 |
| Skeletal muscle mass, kg | 27.3 | ± | 6.3 |  | 30.1 | ± | 6.9 |  | 0.09 |
| Total cholesterol, mg/dL | 204.8 | ± | 32.3 |  | 205.3 | ± | 37.6 |  | 0.95 |
| Triglyceride, mg/dL | 184.0 | ± | 113.5 |  | 196.0 | ± | 84.6 |  | 0.67 |
| ^a^HDL cholesterol, mg/dL | 46.8 | ± | 10.3 |  | 44.7 | ± | 8.6 |  | 0.41 |
| ^b^LDL cholesterol, mg/dL | 134.2 | ± | 30.8 |  | 135.4 | ± | 38.7 |  | 0.89 |
| Fasting blood glucose, mg/dL | 99.2 | ± | 21.0 |  | 113.2 | ± | 49.2 |  | 0.25 |
| Insulin, mIU/mL | 11.6 | ± | 4.5 |  | 11.6 | ± | 4.7 |  | 0.99 |
| Haemoglobin A1c, % | 5.9 | ± | 0.8 |  | 6.2 | ± | 1.5 |  | 0.33 |
| ^c^HOMA-IR | 2.8 | ± | 1.3 |  | 3.2 | ± | 2.1 |  | 0.48 |
| Smoking status |  |  |  |  |  |  |  |  | 0.04 |
| Never | 66 |  | (59.5) |  | 6 |  | (33.3) |  |  |
| Former | 26 |  | (23.4) |  | 4 |  | (22.2) |  |  |
| Current | 19 |  | (17.1) |  | 8 |  | (44.4) |  |  |
| Drinking status |  |  |  |  |  |  |  |  | 0.99 |
| Never | 16 |  | (14.4) |  | 2 |  | (11.1) |  |  |
| Former | 4 |  | (3.6) |  | 0 |  | (0.0) |  |  |
| Current | 91 |  | (82.0) |  | 16 |  | (88.9) |  |  |
| The values are presented as number (percentage) or mean±standard deviation. | | | | | | | | | |
| ^a^HDL: high-density lipoprotein | | | | | | | | | |
| ^b^LDL: low-density lipoprotein | | | | | | | | | |
| ^c^HOMA-IR: homeostatic model assessment of insulin resistance | | | | | | | | | |
| ^d^The *P* values are derived from nonparametric comparison. | | | | | | | | | |

| Supplementary Table 2. The difference of the changes in metabolic parameters across intervention groups. | | | | | | | | | | | | | | | | | |
| --- | --- | --- | --- | --- | --- | --- | --- | --- | --- | --- | --- | --- | --- | --- | --- | --- | --- |
| Variables | Baseline vs. week 6 | | | | |  | Baseline vs. week 12 | | | | |  | Baseline vs. week 24 | | | | |
|  | n | Mean difference± ^h^SE | | | *^i^P* value |  | n | Mean difference± ^h^SE | | | *^i^P* value |  | n | Mean difference± ^h^SE | | | *^i^P* value |
| ^d^SBP, mmHg |  |  |  |  |  |  |  |  |  |  |  |  |  |  |  |  |  |
| ^a^CO | 34 | -8.88 | ± | 2.11 |  |  | 32 | -7.48 | ± | 2.44 |  |  | 33 | -10.95 | ± | 2.09 |  |
| ^b^AO | 38 | -5.09 | ± | 1.65 | 0.16 |  | 35 | -4.84 | ± | 2.30 | 0.43 |  | 36 | -7.29 | ± | 1.83 | 0.19 |
| ^c^APC | 35 | -5.84 | ± | 1.75 | 0.27 |  | 33 | -7.82 | ± | 2.09 | 0.92 |  | 36 | -7.19 | ± | 1.66 | 0.16 |
| ^e^DBP, mmHg |  |  |  |  |  |  |  |  |  |  |  |  |  |  |  |  |  |
| ^a^CO | 34 | -5.53 | ± | 1.45 |  |  | 32 | -4.89 | ± | 1.65 |  |  | 33 | -6.36 | ± | 1.43 |  |
| ^b^AO | 38 | -2.87 | ± | 0.79 | 0.11 |  | 35 | -4.29 | ± | 1.12 | 0.76 |  | 36 | -5.26 | ± | 0.92 | 0.52 |
| ^c^APC | 35 | -4.81 | ± | 1.14 | 0.70 |  | 33 | -5.58 | ± | 1.27 | 0.74 |  | 36 | -5.28 | ± | 1.06 | 0.54 |
| Weight, kg |  |  |  |  |  |  |  |  |  |  |  |  |  |  |  |  |  |
| ^a^CO | 34 | -0.08 | ± | 0.20 |  |  | 32 | -0.10 | ± | 0.26 |  |  | 33 | -0.12 | ± | 0.30 |  |
| ^b^AO | 38 | -0.41 | ± | 0.20 | 0.32 |  | 35 | -0.56 | ± | 0.31 | 0.22 |  | 36 | -0.35 | ± | 0.36 | 0.67 |
| ^c^APC | 35 | -0.63 | ± | 0.28 | 0.13 |  | 33 | -0.98 | ± | 0.46 | 0.04 |  | 36 | -0.96 | ± | 0.37 | 0.08 |
| Body fat mass, kg |  |  |  |  |  |  |  |  |  |  |  |  |  |  |  |  |  |
| ^a^CO | 34 | 0.72 | ± | 0.21 |  |  | 32 | 0.42 | ± | 0.23 |  |  | 33 | 0.13 | ± | 0.34 |  |
| ^b^AO | 38 | 0.11 | ± | 0.28 | 0.09 |  | 35 | -0.17 | ± | 0.34 | 0.15 |  | 36 | -0.64 | ± | 0.38 | 0.13 |
| ^c^APC | 35 | 0.51 | ± | 0.43 | 0.66 |  | 33 | -0.15 | ± | 0.40 | 0.22 |  | 36 | -0.79 | ± | 0.38 | 0.08 |
| Waist circumference, cm | |  |  |  |  |  |  |  |  |  |  |  |  |  |  |  |  |
| ^a^CO | 34 | -0.91 | ± | 0.66 |  |  | 32 | -1.40 | ± | 0.77 |  |  | 33 | -0.08 | ± | 0.96 |  |
| ^b^AO | 38 | -0.78 | ± | 0.83 | 0.90 |  | 35 | -0.86 | ± | 0.75 | 0.62 |  | 36 | 0.79 | ± | 0.91 | 0.51 |
| ^c^APC | 35 | -2.09 | ± | 0.66 | 0.21 |  | 33 | -2.92 | ± | 0.54 | 0.11 |  | 36 | -1.86 | ± | 0.66 | 0.12 |
| Fasting blood glucose, mg/dL | |  |  |  |  |  |  |  |  |  |  |  |  |  |  |  |  |
| ^a^CO | 34 | -0.50 | ± | 1.20 |  |  | 32 | -3.09 | ± | 2.39 |  |  | 33 | -4.97 | ± | 1.94 |  |
| ^b^AO | 38 | -3.24 | ± | 1.61 | 0.37 |  | 35 | -5.43 | ± | 3.21 | 0.85 |  | 36 | -9.31 | ± | 1.41 | 0.21 |
| ^c^APC | 35 | -2.20 | ± | 1.75 | 0.65 |  | 33 | -3.18 | ± | 4.72 | 0.38 |  | 36 | -3.89 | ± | 1.09 | 0.99 |
| ^f^HOMA-IR |  |  |  |  |  |  |  |  |  |  |  |  |  |  |  |  |  |
| ^a^CO | 34 | 0.39 | ± | 0.26 |  |  | 32 | 0.23 | ± | 0.23 |  |  | 33 | -0.11 | ± | 0.16 |  |
| ^b^AO | 38 | 0.13 | ± | 0.21 | 0.46 |  | 35 | -0.28 | ± | 0.19 | 0.07 |  | 36 | -0.39 | ± | 0.15 | 0.37 |
| ^c^APC | 35 | -0.03 | ± | 0.24 | 0.18 |  | 33 | -0.27 | ± | 0.20 | 0.06 |  | 36 | -0.24 | ± | 0.13 | 0.63 |
| Triglyceride, mg/dL |  |  |  |  |  |  |  |  |  |  |  |  |  |  |  |  |  |
| ^a^CO | 34 | -3.32 | ± | 24.16 |  |  | 32 | -48.88 | ± | 25.10 |  |  | 33 | -27.64 | ± | 28.55 |  |
| ^b^AO | 38 | -4.68 | ± | 9.52 | 0.39 |  | 35 | -7.06 | ± | 16.12 | 0.93 |  | 36 | -13.64 | ± | 16.70 | 0.11 |
| ^c^APC | 35 | -14.23 | ± | 10.49 | 0.30 |  | 33 | -0.70 | ± | 15.35 | 0.18 |  | 36 | 9.64 | ± | 10.72 | 0.93 |
| ^g^HDL cholesterol, mg/dL | |  |  |  |  |  |  |  |  |  |  |  |  |  |  |  |  |
| ^a^CO | 34 | -0.91 | ± | 0.95 |  |  | 32 | -0.25 | ± | 1.19 |  |  | 33 | -0.39 | ± | 1.31 |  |
| ^b^AO | 38 | 0.45 | ± | 1.13 | 0.92 |  | 35 | -0.54 | ± | 1.02 | 0.26 |  | 36 | 1.83 | ± | 1.21 | 0.14 |
| ^c^APC | 35 | -0.26 | ± | 1.06 | 0.48 |  | 33 | -1.76 | ± | 1.23 | 0.84 |  | 36 | -0.42 | ± | 1.10 | 0.46 |
| ^a^CO: Control group | | | | | | | | | | | | | | | | | |
| ^b^AO: Application-only group | | | | | | | | | | | | | | | | | |
| ^c^APC: Application with personal coaching group | | | | | | | | | | | | | | | | | |
| ^d^SBP: systolic blood pressure | | | | | | | | | | | | | | | | | |
| ^e^DBP: diastolic blood pressure | | | | | | | | | | | | | | | | | |
| ^f^HOMA-IR: homeostatic model assessment of insulin resistance | | | | | | | | | | | | | | | | | |
| ^g^HDL: high-density lipoprotein | | | | | | | | | | | | | | | | | |
| ^h^SE: standard error | | | | | | | | | | | | | | | | | |
| *^i^P* values are derived from the independent t-test in reference to the control group. | | | | | | | | | | | | | | | | | |

| Supplementary Table 3. Estimated effect of the intervention based on linear mixed model. | | | | | | | | | | | | | | | |
| --- | --- | --- | --- | --- | --- | --- | --- | --- | --- | --- | --- | --- | --- | --- | --- |
| Variables | Baseline vs. week 6 | | |  | Baseline vs. week 12 | | |  | Baseline vs. week 24 | | |  | Continuous change/month | | |
|  | β (^h^SE) | | *^i^P* value |  | β (^h^SE) | | *^i^P* value |  | β (^h^SE) | | *^i^P* value |  | β (^h^SE) | | *^i^P* value |
| ^d^SBP change, mmHg |  |  |  |  |  |  |  |  |  |  |  |  |  |  |  |
| ^a^CO vs. ^b^AO | 3.31 | (2.38) | 0.16 |  | 2.00 | (2.45) | 0.42 |  | 2.93 | (2.42) | 0.23 |  | 0.46 | (0.43) | 0.28 |
| ^a^CO vs. ^c^APC | 2.60 | (2.41) | 0.28 |  | -0.50 | (2.47) | 0.84 |  | 3.18 | (2.42) | 0.19 |  | 0.47 | (0.43) | 0.27 |
| ^e^DBP change, mmHg |  |  |  |  |  |  |  |  |  |  |  |  |  |  |  |
| ^a^CO vs. ^b^AO | 2.42 | (1.51) | 0.11 |  | 0.25 | (1.55) | 0.87 |  | 0.90 | (1.53) | 0.56 |  | 0.06 | (0.27) | 0.83 |
| ^a^CO vs. ^c^APC | 0.96 | (1.53) | 0.53 |  | -0.47 | (1.57) | 0.77 |  | 1.24 | (1.53) | 0.42 |  | 0.19 | (0.27) | 0.47 |
| Weight change, kg |  |  |  |  |  |  |  |  |  |  |  |  |  |  |  |
| ^a^CO vs. ^b^AO | -0.35 | (0.42) | 0.41 |  | -0.60 | (0.43) | 0.16 |  | -0.51 | (0.42) | 0.58 |  | -0.03 | (0.07) | 0.66 |
| ^a^CO vs. ^c^APC | -0.51 | (0.42) | 0.23 |  | -0.93 | (0.43) | 0.03 |  | -0.87 | (0.42) | 0.04 |  | -0.14 | (0.07) | 0.05 |
| Body fat mass change, kg |  |  |  |  |  |  |  |  |  |  |  |  |  |  |  |
| ^a^CO vs. ^b^AO | -0.56 | (0.45) | 0.22 |  | -0.60 | (0.46) | 0.19 |  | -0.73 | (0.46) | 0.11 |  | -0.11 | (0.08) | 0.16 |
| ^a^CO vs. ^c^APC | -0.15 | (0.46) | 0.74 |  | -0.52 | (0.47) | 0.27 |  | -0.95 | (0.46) | 0.04 |  | -0.19 | (0.08) | 0.02 |
| Waist circumference change, cm | |  |  |  |  |  |  |  |  |  |  |  |  |  |  |
| ^a^CO vs. ^b^AO | 0.32 | (0.98) | 0.75 |  | 0.46 | (1.01) | 0.65 |  | 0.99 | (1.00) | 0.32 |  | 0.18 | (0.17) | 0.32 |
| ^a^CO vs. ^c^APC | -0.90 | (1.00) | 0.37 |  | -1.47 | (1.02) | 0.15 |  | -1.59 | (1.00) | 0.11 |  | -0.27 | (0.17) | 0.12 |
| Fasting blood glucose change, mg/dL | |  |  |  |  |  |  |  |  |  |  |  |  |  |  |
| ^a^CO vs. ^b^AO | -1.64 | (2.96) | 0.58 |  | -1.07 | (3.04) | 0.72 |  | -3.05 | (3.01) | 0.31 |  | -0.45 | (0.51) | 0.38 |
| ^a^CO vs. ^c^APC | -2.33 | (3.00) | 0.44 |  | -0.79 | (3.08) | 0.80 |  | 0.43 | (3.01) | 0.89 |  | 0.10 | (0.51) | 0.84 |
| ^f^HOMA-IR change |  |  |  |  |  |  |  |  |  |  |  |  |  |  |  |
| ^a^CO vs. ^b^AO | -0.25 | (0.26) | 0.34 |  | -0.49 | (0.27) | 0.07 |  | -0.29 | (0.26) | 0.27 |  | -0.06 | (0.05) | 0.23 |
| ^a^CO vs. ^c^APC | -0.45 | (0.26) | 0.09 |  | -0.55 | (0.27) | 0.04 |  | -0.22 | (0.26) | 0.40 |  | -0.04 | (0.05) | 0.32 |
| Triglyceride change, mg/dL |  |  |  |  |  |  |  |  |  |  |  |  |  |  |  |
| ^a^CO vs. ^b^AO | -17.01 | (21.09) | 0.42 |  | 23.06 | (21.73) | 0.29 |  | -0.04 | (21.46) | 0.99 |  | 1.45 | (3.71) | 0.70 |
| ^a^CO vs. ^c^APC | -32.69 | (21.44) | 0.13 |  | 23.28 | (21.99) | 0.29 |  | 17.04 | (21.46) | 0.43 |  | 4.47 | (3.68) | 0.23 |
| ^g^HDL cholesterol change, mg/dL | |  |  |  |  |  |  |  |  |  |  |  |  |  |  |
| ^a^CO vs. ^b^AO | 1.73 | (1.36) | 0.20 |  | 0.06 | (1.40) | 0.97 |  | 2.52 | (1.38) | 0.07 |  | 0.35 | (0.24) | 0.14 |
| ^a^CO vs. ^c^APC | 0.75 | (1.38) | 0.59 |  | -1.05 | (1.41) | 0.46 |  | 0.16 | (1.38) | 0.91 |  | -0.03 | (0.24) | 0.91 |
| ^a^CO: Control group | | | | | | | | | | | | | | | |
| ^b^AO: Application-only group | | | | | | | | | | | | | | | |
| ^c^APC: Application with personalised coaching group | | | | | | | | | | | | | | | |
| ^d^SBP: systolic blood pressure | | | | | | | | | | | | | | | |
| ^e^DBP: diastolic blood pressure | | | | | | | | | | | | | | | |
| ^f^HOMA-IR: homeostatic model assessment of insulin resistance | | | | | | | | | | | | | | | |
| ^g^HDL: high-density lipoprotein | | | | | | | | | | | | | | | |
| ^h^SE: standard error | | | | | | | | | | | | | | | |
| *^i^P* values are derived from the independent t-test in reference to the control group. | | | | | | | | | | | | | | | |
