# Supplementary figures and images for: Effect of Smartphone-Based Lifestyle Coaching App on Community-Dwelling Population With Moderate Metabolic Abnormalities: Randomized Controlled Trial
Source: J Med Internet Res. 2020 Oct 9;22(10):e17435. doi: 10.2196/17435 (PMC7584978; doi:10.2196/17435)

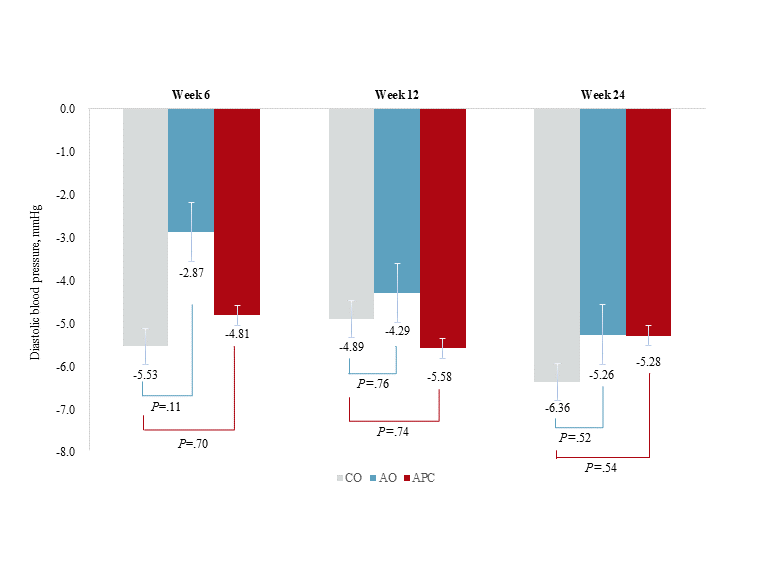

Supplement: Multimedia Appendix 2 [file jmir_v22i10e17435_app2.png]

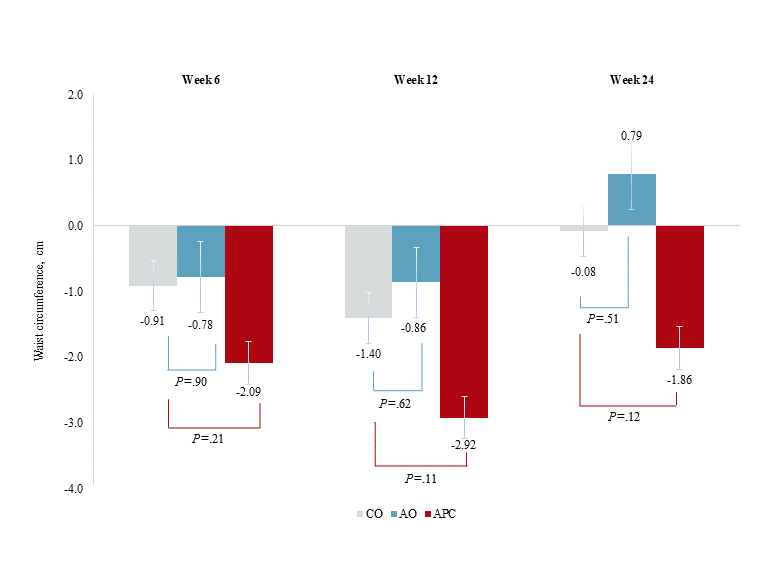

Supplement: Multimedia Appendix 3 [file jmir_v22i10e17435_app3.png]

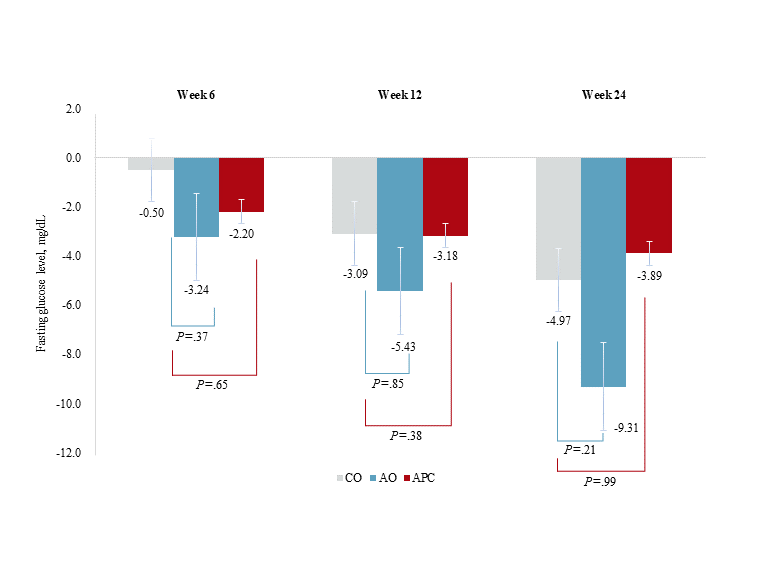

Supplement: Multimedia Appendix 4 [file jmir_v22i10e17435_app4.png]

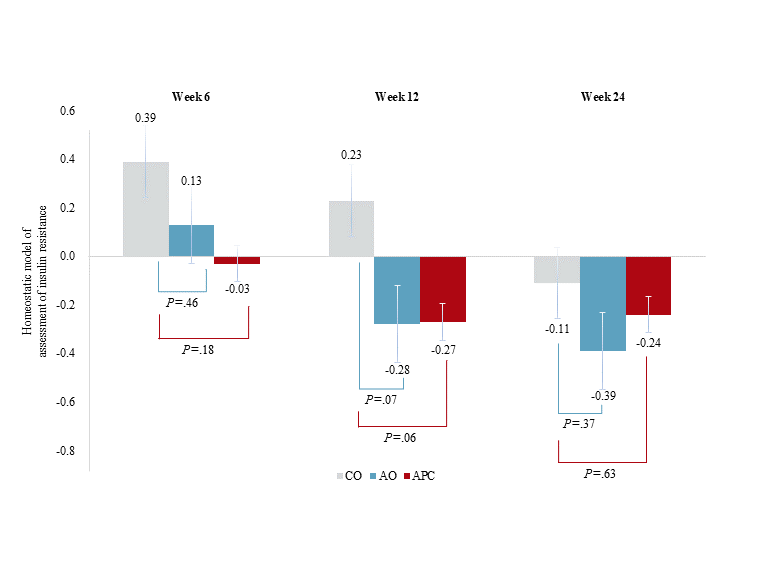

Supplement: Multimedia Appendix 5 [file jmir_v22i10e17435_app5.png]

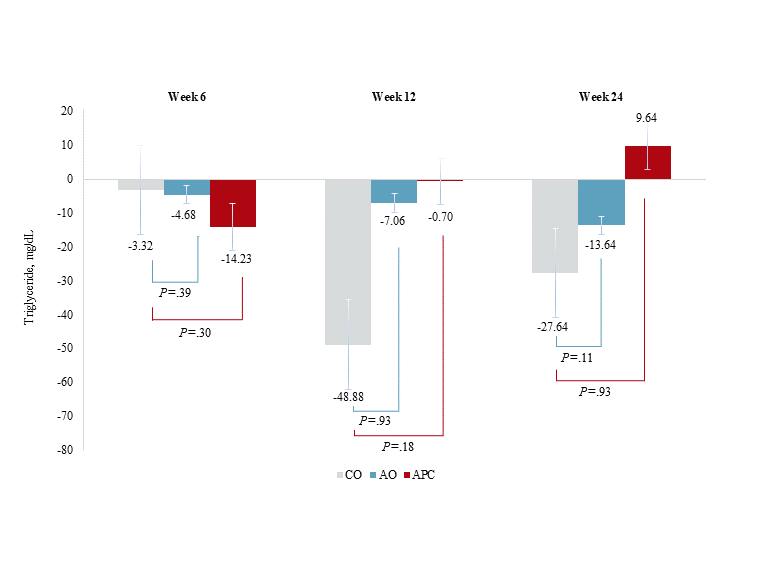

Supplement: Multimedia Appendix 6 [file jmir_v22i10e17435_app6.png]

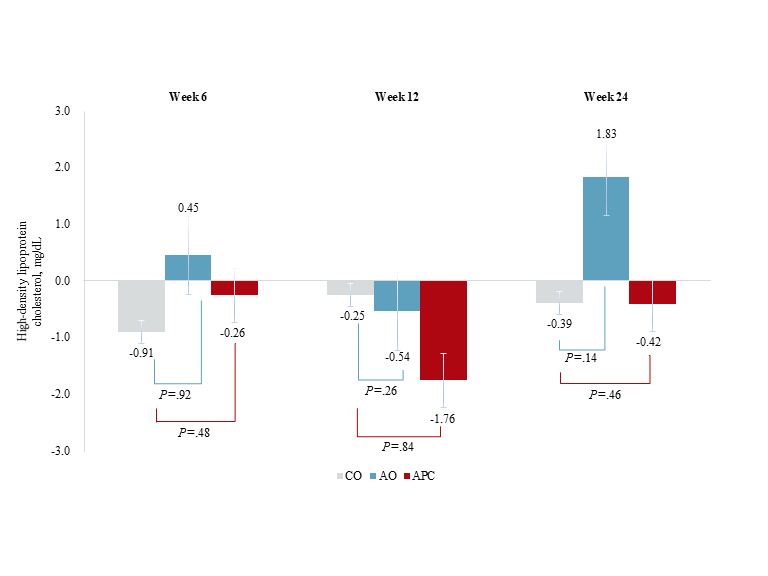

Supplement: Multimedia Appendix 7 [file jmir_v22i10e17435_app7.png]
